# Comment on Ramai et al. Risk of Hepatocellular Carcinoma in Patients with Porphyria: A Systematic Review. *Cancers* 2022, *14*, 2947

**DOI:** 10.3390/cancers15030795

**Published:** 2023-01-28

**Authors:** Mattias Lissing, Daphne Vassiliou, Pauline Harper, Eliane Sardh, Staffan Wahlin

**Affiliations:** 1 Hepatology Division, Department of Upper GI Diseases, Karolinska University Hospital, 17164 Stockholm, Sweden; 2 Department of Medicine, Huddinge, Karolinska Institutet, 14186 Stockholm, Sweden; 3 Department of Endocrinology, Karolinska University Hospital, 17164 Stockholm, Sweden; 4 Centre for Inherited Metabolic Diseases (CMMS), Porphyria Centre Sweden, Karolinska University Hospital, 17164 Stockholm, Sweden; 5 Department of Molecular Medicine and Surgery, Karolinska Institutet, 14186 Stockholm, Sweden; 6 Department of Medical Biochemistry and Biophysics, Karolinska Institutet, 14186 Stockholm, Sweden

We read with interest this review by Ramai et al. [1], who compiled an impressive number of studies with the aim of assessing the risk of hepatocellular carcinoma (HCC) in patients with porphyria. This review is a commendable attempt to explore this important research area, but unfortunately it includes so many misinterpretations, mix-ups, and errors that we feel compelled to comment.

First some background for context: It is well known that patients with two different types of porphyria, acute intermittent porphyria (AIP) and porphyria cutanea tarda (PCT) have an increased risk of primary liver cancer (PLC), mainly HCC. PCT, the most common type of porphyria, is in most patients an acquired enzyme deficiency caused by underlying liver diseases such as hepatitis C or alcohol-related liver disease. A minority have an inherited enzyme deficiency that causes a disease with similar clinical manifestations. AIP, variegate porphyria (VP), and hereditary coproporphyria (HCP) are commonly grouped together as acute hepatic porphyrias (AHP) due to similar clinical and biochemical features. The AHP are inherited monogenic diseases with low penetrance that affect different enzymes in the heme synthesis pathway and potentially lead to the accumulation of the heme precursors delta-aminolaevulinic acid (ALA) and porphobilinogen (PBG).

AHP and PCT are completely different diseases, and the carcinogenic pathways differ significantly. While patients with PCT have an underlying liver disease that may lead to an increased risk of cirrhosis and PLC, AHP patients rarely have significant liver fibrosis. Most AHP-associated PLCs are diagnosed in non-cirrhotic livers and the current understanding is that the carcinogenesis pathway is related to the intrahepatic accumulation of ALA [2,3,4]. Several previous studies have demonstrated a high risk of PLC in AHP, but the risk estimates vary depending on the composition of the studied cohorts. The field has several areas that need elucidation. Further studies are needed to assess the risk of PLC in HCP and VP. Determining potential individual PLC risk factors in patients with AHP would help to identify high risk groups who would benefit from surveillance the most.

We have several comments on the contents of the review.

To start with, the authors chose to include studies on both PCT and AHP in this review: 10 studies include only AHP, 7 only PCT, and 2 studies include both AHP and PCT patients. If doing so, the cohorts must be assessed, analyzed, and discussed separately. To combine cohorts with AHP and PCT, and to only present the common risk estimate is comparable to combining patients with, for instance, viral and autoimmune hepatitis, two diseases with fundamentally different pathogenesis, and only presenting the combined risk estimate.

It is hard to follow which patient group that the authors refer to in different parts of this review. The vague term “porphyria” is used alternately with the more precise AHP in an imprecise and, in some instances, erroneous way. Two examples:

- In the first statement of the discussion, the authors state that “patients with AHP had an estimated 5% risk of primary liver cancer”. This is incorrect, since the 5% risk estimate reflects the combined risk of the AHP and PCT cohorts. The risk in AHP is not assessed in this review.
- In the final paragraph of the discussion, the authors write that “This study provides further evidence to the current understanding of HCC in patients with AHP. This extensive review includes 19 studies from multiple countries, supporting screening guidelines in this patient population”. Again, only the term AHP is used. Only 12 of those 19 studies included patients with AHP, which makes the reference to AHP incorrect. Furthermore, PCT, the porphyria type included in nine of the studies, is not mentioned at all in the discussion. It is unclear whether the authors included the PCT studies erroneously as AHP, or if those studies were in fact not included in the final conclusions.

Neither age nor disease severity are included in the analysis or the discussion, in this study, which makes the risk assessment less accurate. Cohorts included in this review vary significantly in terms of demographics and clinical disease severity. Age is a significant risk factor for the development of HCC [5]. As an example, the cohort in the Innala study (mean age 67, high proportion of clinically active disease) and the Andant cohort (mean age of 41) cannot be compared without accounting for the difference in the age of the included patients [6,7]. Differences in disease severity, assessed as clinically or biochemically active disease vs. asymptomatic gene carriers are important, as it has been shown to be a major risk factor in AHP [8,9]. Most AHP gene carriers are asymptomatic, and they have little or no biochemical activity [10,11]. Others have frequent attacks and are more likely to be under medical attention, included in registers and offered follow-up. This constitutes a high risk of surveillance bias. For studies on PCT cohorts, the proportion and severity of underlying liver disease must be considered, as this is the single most important predictor of the risk for HCC. Unfortunately, this aspect was not assessed, nor addressed in the discussion section.

The main aim of this review was to assess the PLC risk in “porphyria”. Including both PCT and AHP is, as mentioned above, of doubtful relevance. The results nevertheless deserve a thorough examination. The stated 4.8% risk of PLC in “porphyria” was calculated by dividing the total number of patients with PLC (351) by the total number of patients at risk (7381). That estimation is based on incorrect calculations.

Starting with the numerator, there are significant errors. The authors included 102 cases of PLC from the study by Hardell et al. from 1984 [12]. This case-control study included 103 individuals who died from liver cancer, 9 of whom had porphyria. When patients without porphyria are subtracted, the total number of individuals is 262, rather than 351. The numerator is thus overestimated by at least 93 (35%) cases. This fallacy is repeated in the Abstract, Results, and Discussion sections.

We also object to the authors’ approach to the issue of overlapping studies. In the methods, the authors state that “In the event of multiple publications from the same cohort or overlapping cohorts, data from the most recent and/or most appropriate comprehensive report were retained.” This is an important principle that was unfortunately not followed throughout this review.

The important study by Baravelli et al. from 2017 [8] on PLC risk in AHP was excluded because another publication from the same group on PCT and cancer risk was published 2 years later [13]. These are, however, two completely different cohorts with no overlap whatsoever. The study by Baravelli should obviously have been included.

Many of the studies from Sweden have significant overlap but none were excluded. For example: Lithner 1984 [14] overlaps with Hardell 1984 [12]. Lissing 2021 [9] includes all Swedish patients 1987–2015, and therefore overlaps with Sardh 2013 [15], Elder 2013 [16], Innala 2011 [7], Linet 1999 [17], and Andersson 1996 [18]. The study of Andersson 1996 [18] includes all cases from the northernmost county in Sweden 1978–1990, and thus overlaps with Lithner 1984 [14]. Also, some of these studies overlap more than twice. For example, some HCC cases in Elder 2013 [16] are included in Sardh 2013 [15] and Lissing 2021 [9]. Many PLC cases from these studies are thus counted more than once, which makes the final result even more overestimated.

Continuing with the denominator (*n* = 7381), patients at risk, we again find several significant errors:

- The 103 cases in the Hardell study [12] are considered as the total cohort number. This is incorrect. In this case-control study, the population at risk was unknown and not mentioned in the original paper, and can therefore not be used to calculate incidence.
- According to Table 1 in the review [1], 335 cases from the Elder 2013 study [16] are included. This is incorrect. The Elder study was designed to assess the incidence and prevalence of different types of porphyria; 335 was the number of new cases of porphyrias in Europe during the study period, and not the study population from which the HCC cases were identified. No cohort was defined, and the cumulative incidence can therefore not be calculated from this study.
- The authors state that the porphyria cohort size in the study by Andersson 1996 [18] was 2122. This is highly inaccurate. The prevalence of AIP is high in the northernmost part of Sweden, but the total national number of AIP cases in Sweden is not more than approximately 1000 individuals [19]. The reported number (2122) represents all of the inhabitants who died in the studied municipalities during the study period. The AHP population size in the municipalities included is not mentioned in the original paper, but it is probably less than 300. Based on this error alone, the sum of individuals included in the denominator is overestimated by more than 1800.
- Concerning the Innala 2011 study [7], the stated study cohort size is 81, but this only represents one part of the cohort. The total cohort size was 180, clearly stated in the original paper.
- Concerning the Lang 2015 study [20], the stated study cohort size (122) is incorrect. The data on cancers were only available in 49 individuals, clearly stated in the original paper.

The main results of this review are thus not valid. The calculated numbers of included patients with PCT and AHP, porphyria-related PLC, and cumulative incidence of PLC in porphyria are based on a series of incorrect assessments.

Some of the points made in the discussion and conclusions also merit a few comments. The authors state in the results that “not all cases of HCC were preceded by advanced liver fibrosis or cirrhosis”, and a similar statement is made in the discussion. This is well known for AIP, as most AIP-related HCC cases develop in non-cirrhotic livers [3,15]. HCC in PTC is however, commonly associated with cirrhosis in most patients. This difference is an important distinction that should have been made in this paper.

ALA and PBG levels are of great interest as possible indicators of PLC risk in AHP, and new data would be much appreciated. ALA and PBG, as well as porphyrins, are mentioned in the discussion, but not at all in the results or methods. No analysis regarding ALA or PBG as predictors was performed. In Table 1 in the review [1], one column is labeled “urinary cut-offs”, but it is unclear as to what this represents. For some studies, e.g., Innala 2011 [7], Sardh 2013 [15], and Lissing 2021 [9], this column appears to represent the upper limit of normal, while the numbers presented for Andant 2000 [6] are mean ALA and PBG values in patients with PLC. This column thus presents completely different types of information without any clarification or interpretation. Unfortunately, no conclusions can be drawn about any possible associations between elevated ALA and PBG, and an increased risk of PLC, as no results are presented. Also, the suggested link between ALA and carcinogenicity, is only relevant in AHP. The authors incoherently refer to several PCT studies on this matter. In the published response to reviewer 1, the authors claim that the “urinary cut-offs” column describes “thresholds to ensure maximal detection of abnormal patients. All of the values above the cut-off are considered positive”. This is, at least for the Andant study [6], not accurate.

Alpha-fetoprotein (AFP) is of interest as a possible tool in HCC surveillance. The EASL guidelines [5] do not recommend AFP in a surveillance of cirrhotic patients, since both sensitivity and specificity are insufficient. In this paper, the authors mention AFP in the Introduction, which would be an interesting aspect of this review. Some data are presented in Table 1 in the review [1], but no further analysis on how AFP performs as a predictor of HCC in porphyria is presented in the Results section or elsewhere. Without any analysis, the authors state in the discussion that “AFP levels were found to be elevated”, and that “AFP should be used in conjunction with imaging modalities to screen for HCC”. This is a completely new recommendation that warrants more robust analysis and data prior to publishing. According to Table 1 [1], 24 (7%) of the stated 351 patients with porphyria associated PLC had elevated AFP. It cannot be considered serious to suggest surveillance based on a test with such limited sensitivity. Whether AFP might have prognostic capacities in subgroups of patients with porphyria is unclear. It was not assessed in this review.

Finally, in the results (Table 2) and in the discussion, the authors state that 56 (or 48, according to the abstract) liver transplantations were performed with PLC as an indication, referring to Elder et al. [16]. This is false and should have struck both authors and reviewers as an astonishing number, considering that the study only included 14 patients with cancer. In the original paper, the number 56 refers to patients with acute hepatic porphyria with a severe disease phenotype. Three of these fifty-six received transplants during the three years of the study observation time (two in the United Kingdom and one in Sweden), and none of them had PLC. In our study on LT in patients with AIP, we assembled all 38 known transplantations in Europe [21]. Two of the transplanted patients had PLC as part of the LT indication.

A list of additional comments regarding this review are presented in Table 1 below.

Considering the lack of fact-checking, the amount of inaccurate data included in the analyses, and the poor understanding of the different disease entities included in this review, we suggest a thorough revision of this paper. We strongly recommend including researchers with porphyria experience in the revision process, and to invite reviewers with such expertise to ensure a rigorous peer-review process. In its present form, this review by Ramai et al. misleads the readership.

## Figures and Tables

**Table 1:** Additional comments on specific statements in the review.

Section	Stated Text	Comment
Abstract (similar in the simple summary and introduction)	Subclinical liver disease is common, which can progress into transaminitis, fibrosis, cirrhosis, and malignancy	Although this is interesting, stating that liver disease is common and implying that fibrosis and cirrhosis are common in all porphyrias should be supported by evidence.
Abstract	Its [the porphyrias] estimated prevalence nears 5 per 100,000 patients worldwide	The prevalence of the porphyrias cannot be generalized. The group includes ultra-rare porphyrias with only a few cases described in the world literature (ALAD-porphyria), rare porphyrias with a few hundred known cases worldwide (congenital erythropoietic porphyria), and those with unknown prevalence (X-liked erythropoietic porphyria). Of the types of porphyrias that are the focus of this review, the prevalence of PCT is 1/10,000 to 1/25,000, and the prevalence of AHP varies between different populations. The reference used is referring to a paper that refers to a book chapter based on outdated expert opinions. Our advice is to use epidemiological data from the Elder study [15]. Furthermore, the question of prevalence in porphyria is complex. Are all gene-carriers included, or only subjects with symptomatic disease? Definitions of what constitutes disease and of porphyria type are necessary in statements on prevalence and incidence.
Abstract	“Porphyrias are inborn defects”	Most patients with PCT have the acquired form, not the inherited form.
Introduction, third paragraph	“A recent European study reported an annual incidence of HCC 0.07%” and “would warrant surveillance in non-cirrhotic patients, supporting a rationale for an active surveillance program in this cohort”	The study [13] that the authors refer to actually states the opposite: “Therefore, surveillance cannot be currently recommended based on a PCT diagnosis alone.”
Introduction	Acute hepatic porphyrias (AHP) are a group of four ultra-rare metabolic disorders	A disease is generally considered to be ultra-rare if it affects one patient per 50,000 people (or, fewer than 20 patients per million of population), and most ultra-rare diseases affect much fewer. Some porphyrias are considered ultra-rare (ALAD-porphyria for instance), but AHP (AIP, VP, and HCP) and PCT are not.
Results, Table 1	Porphyria subtype with liver cancer (column)	The percentage of porphyria subtypes vary between <1% and 88%. If true, this would be a significant finding. However, this column includes a random mix of cumulative incidences (numbers of patients with PLC/numbers at risk) and percentage of patients with PLC and a specific type of porphyria/total number of patients with PLC (e.g., Kauppinen 1992, Andersson 1996, Sardh 2013). The same way of presenting numbers should be used throughout this column.
Results, Table 1	Linet et al. Location: Denmark	This study was performed in Sweden and Denmark. All PLC cases were identified in Sweden [17].
Results, Table 1	Andant et al. Location: Italy	This study was performed entirely in France [6].
Results, Table 1	Elder et al. Design Retrospective. Location France.	This was a prospective study performed in a collaboration by 11 European Porphyria Centers [15]. France was indeed one of these, but no cases of liver cancer were reported from France; 11 were from Sweden and one each from the Netherlands, the United Kingdom, and Switzerland.
